# Embolization of uterine arteriovenous malformation

**Published:** 2013-02

**Authors:** Yan Chen, Guoyun Wang, Fubo Xie, Bo Wang, Guowei Tao, Beihua Kong

**Affiliations:** 1 *Department of Obstetrics and Gynecology, Qilu Hospital of Shandong University, Jinan 250012, China.*; 2 *Department of Interventional Radiology, Qilu Hospital of Shandong University, Jinan 250012, China.*; 3 *Department of Ultrasonography, Qilu Hospital of Shandong University, Jinan 250012, China.*

**Keywords:** *Uterine arteriovenous malformation*, *Doppler ultrasonography*, *Angiography*, *Therapeutic embolization*

## Abstract

**Background: **Uterine arteriovenous malformation is a rare but potential life-threatening source of bleeding. A high index of suspicion and accurate diagnosis of the condition in a timely manor are essential because instrumentation that is often used for other sources of uterine bleeding can be lead to massive hemorrhage.

**Case: **We describe here a case of uterine arteriovenous malformation. A 32-year-old woman presented abnormal vaginal bleeding following the induced abortion. A diagnosis of uterine arteriovenous malformation made on the basis of Doppler ultrasonraphy was confirmed through pelvic angiography. The embolization of bilateral uterine arteries was performed successfully.

**Conclusion:** Uterine arteriovenous malformation should be suspected in patient with abnormal vaginal bleeding, especially who had the past medical history incluing cesarean section, induced abortion, or Dillation and Curethage and so on. Although angiography remains the gold standard, Doppler ultrasonography is also a good noninvasive technique. The transcatheter uterine artery embolization offers a safe and effective treatment

## Introduction

Uterine arteriovenous malformation (AVM) is a rare but potentially life-threatening source of bleeding. Dubreil and Loubat described the first clinical case involving a uterine AVM in 1926. AVMs have been reported in patients from 18 to 72 years old but only rarely in nulliparous women (1). Uterine AVMs are characterized by multiple communications of varying sizes between arteries and veins in the same vicinity. Uterine AVMs have been classified as congenital or acquired (2). 

Congenital uterine AVMs arise from an abnormality in the embryological development of primitive vascular structures, resulting in multiple abnormal communications between arteries and veins (2). Acquired uterine AVMs are usually traumatic, resulting from prior dilation and curettage (D&C), uterine surgery, or direct uterine trauma, and less commonly from endometrial carcinoma, cervical carcinoma, and gestational trophoblastic disease. Acquired AVMs are small arteriovenous fistulas between intramural arterial branches and the myometrial venous plexus. 

They appear as a vascular tangle (3, 4). The classical presentation of uterine AVMs is often one of severe uterine bleeding with no obvious cause. The onset and cessation of bleeding are abrupt, comparable to the opening and closing of a faucet. Other symptoms include lower abdominal pain, dyspareunia, and anemia secondary to blood loss. In very severe AVMs, shunting can cause cardiovascular repercussions, provoking symptoms of dyspnea, fatigue, and even heart decompensation (5, 6). Signs such as an audible bruit, a palpable thrill in the groin, or a pulsating mass on manual examination have been observed (7, 8), and edematous lower extremities due to venous stasis may be seen (7). 

Several imaging methods, such as Doppler ultrasonography, computed tomography, magnetic resonance imaging (MRI), and angiography, have been employed to diagnose AVMs. Angiography is the gold standard for diagnosis, whereas Doppler ultrasonography and MRI are the modalities of choice for the evaluation of a suspected AVM. Ultrasonography and MRI can not only define a uterine AVM accurately but also assess the extent of pelvic involvement non-invasively. 

Prior to embolotherapy, conservative treatment, such as expectant management and medication, hysterectomy or uni/ bilateral uterine artery ligation, were the therapies of choice. Embolotherapy, which offers the major advantage of maintaining childbearing capacity, has become a well-recognized alternative to surgical intervention for treating uterine AVMs since the first reported case of transcatheter uterine artery embolization for uterine AVM in 1982. 

The current case report presents a patient with AVM initially diagnosed by color Doppler imaging, confirmed by angiography, and finally treated by transcatheter uterine artery embolization.

## Case report

A 32-year-old woman, gravida 3, para 2, suffering from abnormal vaginal bleeding for approximately 20 days 2 months after her gestational 4-month induced abortion, was admitted for emergency treatment due to syncope on exertion. She had undergone a vaginal delivery 9 years prior to admission and Caesarean section 9 months earlier. No history of excessive bleeding during or after the vaginal delivery or Caesarean section was noted. Her menstrual cycles were regular after menarche, at an interval of 30 days, and the bleeding lasted for about 5 days. 

No dysmenorrhea was reported. Upon admission to the hospital, the patient received blood transfusion and fluid infusion. General examination of the woman was normal except for a severely anemic appearance. Pelvic examination showed the uterus to be slightly enlarged and feebly tender, and no adnexal abnormality on palpation was observed. The vulva and cervix were also normal. At this point, the patient’s hemoglobin level was 5.5 g/dL and her serum beta-human chorionic gonadotropin (β-hCG) was 1.13 mIU/mL. On a gray-scale image, a 33 mm×27 mm ill-defined hypoechoic lesion was observed on the anterior wall of the uterus, which was prominent towards the uterine cavity. 

**Figure 1:**
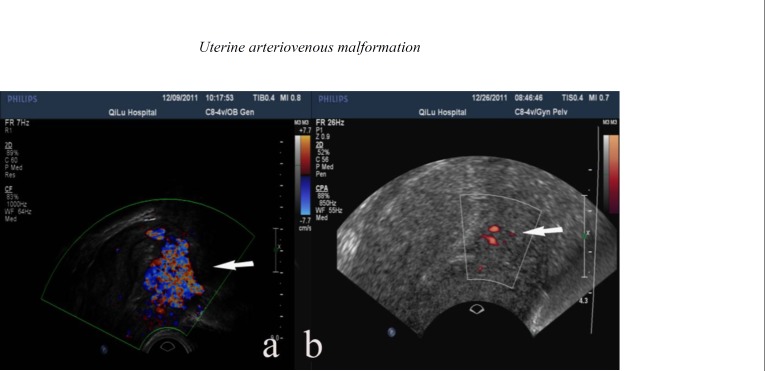
Color Doppler imaging features of uterine arteriovenous malformation (AVM) pre- and post- embolization. (a) Color Doppler image showing mosaic pattern and turbulent flow (arrow) pre-embolization. (b) Color Doppler image two weeks after embolization indicating markedly reduced blood flow (arrow

Color Doppler sonography demonstrated hypervascularity throughout the described lesion, and a color mosaic pattern represented a turbulent flow (Figure 1a). Spectral analysis of the arterial vessels showed a high-velocity (peak systolic velocity (psv) of 45 cm/s), low-resistance (resistance index (RI) of 0.34) flow.

**Figure 2:**
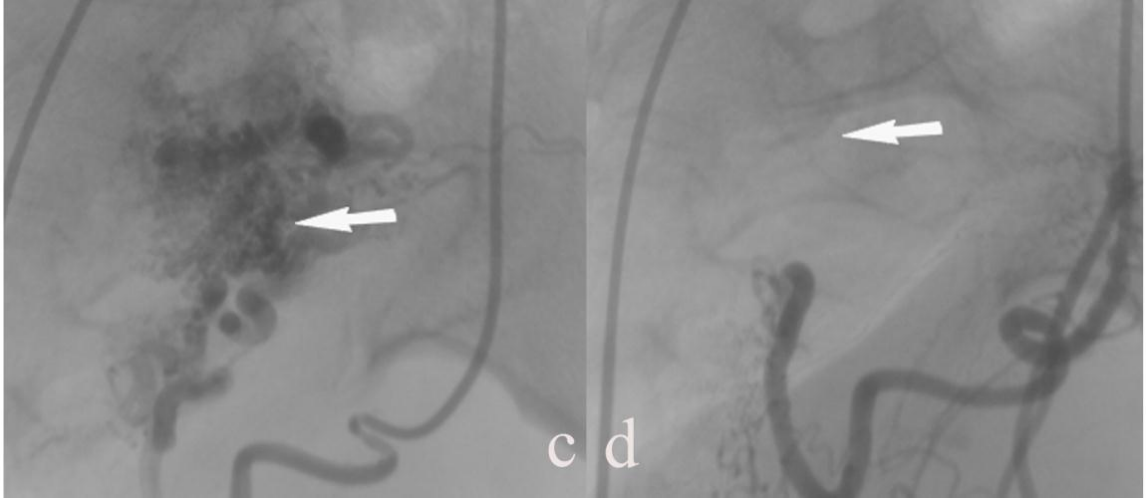
Arteriograms pre- and post- eembolization. (c) Arteriogram pre-embolization showing a tangle of vascular structures (arrow) fed by the left uterine artery. (d) Arteriogram post-embolization showing the absence of filling of the occluded left uterine artery and vascular malformation (arrow

These findings were most suggestive of uterine AVM. In addition, the color sonographic findings of the ovaries were normal. The patient was referred to interventional radiology for pelvic angiography and uterine artery embolization of uterine AVM. After informed consent was obtained, a regional anesthetic technique was performed. The right common femoral artery was accessed, and a 5F glide catheter was placed through a 5F sheath. Contrast injection through a catheter in the left internal iliac artery demonstrated a tangle of vascular structure fed primarily via the left uterine artery (Figure 2c). A 5F selective catheter was placed into the left uterine artery. Contrast injection demonstrated a serpiginous and dilated arterial structure. Gelfoam pledgets were injected into the left uterine artery until near stasis of flow was encountered. 

The 5F catheter was then withdrawn and placed in the right internal iliac artery. Contrast injection through a catheter in the right internal iliac artery revealed a tangle of vascular structures fed by the right uterine artery. These indicated that the AVM was fed by bilateral uterine arteries. As performed above, the 5F selective catheter was then withdrawn and placed into the right uterine artery. Contrast injection showed a tortuous vascular mass. Gelfoam pledgets were injected into the right uterine artery until near stasis of flow. After bilateral uterine artery embolization, angiograms revealed the obliteration of uterine AVM (Figure 2d).

The patient experienced a mild lower abdominal discomfort for several days after the procedures but made an uneventful recovery. A repeat gray-scale imaging performed two weeks later suggested a 12 mm × 9 mm hypoechoic lesion in the anterior wall of the uterus that had a significant change in the appearance of the vascular malformation. In addition, a markedly reduced blood flow pattern was observed on the color Doppler ultrasound (Figure 1b). The menstrual cycle of the patient returned to normal one month after follow-up. An ongoing follow-up was conducted.

## Discussion

Uterine AVMs are uncommon, and their true incidence is unknown. AVMs are isolated anomalies in otherwise healthy persons and are particularly variable in size and anatomic location (9). As a normal blood vessel courses towards the region of the tissue it supplies, it divides into smaller branches. By the time it reaches its destination, it would have branched into many thin capillaries. 

The blood flow within AVM is slow and under low pressure because many capillaries are normally present. Nevertheless, an AVM consists of a proliferation of vascular channels with fistula formation and an admixture of small, capillary-like channels. Congenital AVMs tend to have multiple feeding arteries, draining veins, and intervening nidus, whereas acquired AVMs tend to have single or bilateral feeding arteries, are not supplied by extrauterine arteries, and do not have a nidus (10, 11). 

Given these different structural characteristics, acquired AVMs are easier to treat by transcatheter arterial embolization than congenital AVMs (10). Coupled with imaging findings, patient history helps differentiate between acquired and congenital AVMs. The AVM in the present case report was assumed to be acquired because of the patient’s history, which includes a Caesarean section and induced abortion. 

Uterine AVMs result in sudden and massive vaginal bleeding that may be life-threatening, suggestive of arterial hemorrhage (4). Uterine bleeding is thought to occur when the vessels of the AVM are exposed from iatrogenic sloughing of the endometrium during D&C or during the menstrual period (2). For patients who almost have moderate to severe vaginal bleeding, procedures such as D&C should be considered with caution or even contraindicated because fatal bleeding after these would occur in patients with true AVM. Consequently, prompt, accurate diagnosis is crucial for good patient outcomes. 

In the past, diagnosis was made after hysterectomy and histopathological examination. Currently, angiography is the gold standard for diagnosis (12). Although a definitive diagnosis is usually made by pelvic angiography, Doppler ultrasonography provides a valuable, non-invasive method of diagnosing uterine AVMs. Gray-scale ultrasonography itself may play a role in the diagnosis but the features of AVMs may be similar to other pelvic structures or pathologies (13). 

The addition of color Doppler improves the diagnostic ability of ultrasonography (14). A localized area of increased vascularity within the myometrium itself typifies these lesions. Pulsed Doppler evaluation of the identified area will normally reveal a low-resistance blood flow with high peak velocities and evidence of turbulence (2). The waveform is usually broad with an irregular spectral envelope, indicating a turbulent flow resulting from the many direct arteriovenous connections that are present. 

Continuous high blood flow throughout both the systolic and diastolic components of the cardiac cycle is usually observed. Analysis of the waveform will show a typically high PSV with low values of RI and PI. Doppler examination should be conducted prior to D&C, which should be avoided by women with AVMs because the procedure may worsen bleeding. MRI provides an accurate definition of uterine AVMs and effectively delineates the invasion of adjacent organs. The characteristic features include a bulky uterus with a focal mass, disruption of the junctional zones, multiple serpiginous flow-related signal voids within the lesion, and prominent parametrial vessels (2, 12). 

Gadolinium-enhanced MRI demonstrates a hypervascular arterial-dominant flow. Similar to MRI, computed tomography (CT) may be used to determine the size, extent, vascularity, and involvement of the adjacent organs (13, 15). In angiographs, the affected arteries appear thicker and more circuitous than normal ones. AVMs appear as a complex tangle of vessels supplied by enlarged feeding arteries and show early venous drainage during the arterial phase (13). Angiography, an invasive technique, allows the confirmation of the diagnosis and helps identify the leading feeder vessels where embolization may be indicated as a conservative treatment option (10). 

Several cases of AVMs have been found during hysteroscopy, but their value is limited (4). Uterine AVMs should be differentiated from the retained products of conception, gestational trophoblastic disease, dysfunctional uterine bleeding, subinvolution, hemangiomas, varicosities, and malignancies of the uterus, such as sarcomas. When the clinical history, ultrasonographic findings, and serum β-hCG test results are considered, AVMs can be differentiated potentially from these pathologic conditions with an arteriovenous shunt (2). Meanwhile, overdiagnosis of uterine AVMs should be avoided (15).

Kido *et al* reported on a patient who presented with sudden, heavy vaginal bleeding 6 weeks after an induced abortion. Gray-scale sonography showed several serpiginous and tubular anechoic spaces within the myometrium, and color Doppler imaging revealed hypervascularity, marked turbulence, and low-impedance, high-velocity flow within the lesion, suggesting a uterine AVM (16). 

MRI findings also suggested an AVM and β-hCG serum levels were slightly elevated. The presumptive diagnosis of a uterine AVM and severe hemorrhage persisted; consequently, hysterectomy was performed in the patient. A pathological examination showed retained placental products that were necrotic and encroached into the myometrium (16). This example illustrates that accurate or precise initial diagnosis and differential diagnosis should be made to prevent overdiagnosis of uterine AVMs.

Traditionally, hysterectomy or uterine arteries ligation were the treatment modalities for cases of uterine AVMs. Angiographic arterial embolization has recently become the preferred management protocol because it is minimally invasive and has the potential to preserve fertility. Several authors have described the regression of AVM with conservative therapy or spontaneous resolution (17). Women with a single episode of bleeding and who were hemodynamically stable were treated expectantly or medication. Some women become asymptomatic with time, suggesting that traumatic AVMs may regress spontaneously. 

In stable women, expectant management may play a role in the regression of AVMs. Timmerman *et al* showed that of 265 patients with abnormal premenopausal bleeding, 9 had uterine AVMs diagnosed on ultrasonography (17). Of these 9 cases of uterine AVM, 6 had spontaneous resolution, 2 patients with hydatidiform mole needed chemotherapy (the AVMs resolved after chemotherapy), and only one required embolization. Long-term medical management may be used if the bleeding is not severe and if the treatment plan includes estrogens and progestins, methylergonovine, danazol, 15-methyl-prostaglandin F2alpha, oral contraceptives, and intramuscular followed by oral methylergonovine maleate (4, 7). 

Recently, a report by Montanari and Alfei presented an AVM patient who was treated with intravenous conjugated estrogens and oral methylergometrine maleate (18). The bleeding regressed on the fourth day of therapy. Initial color Doppler ultrasound examination showed a large amount of turbulent arterial blood flow in the AVM, which was normalized with the resolution of symptoms. Methylergonovine maleate was thus suggested to induce tetanic myometrial contractions and reduce blood flow to the AVM, causing it to collapse; intravenous conjugated estrogens help by covering the hemorrhaging vessels with a proliferative endometrium.

If episodes of recurrent bleeding occur, or if a woman experiences severe bleeding or become hemodynamically unstable, angiography embolizatin is considered (17). Transcatheter arterial embolization has emerged as a highly effective technique for controlling obstetric and gynecologic hemorrhages and has revolutionized the management of uterine AVMs. The size of the AVMs in imaging studies does not correlate with the need for embolization; this decision relies entirely on the clinical condition of the patient (19). 

An atypical embolization procedure is as follows: Using the Seldinger technique through the common femoral artery, initial pelvic angiography is performed followed by selective internal iliac angiography and uterine angiography on the side presumed to be affected during ultrasonographic examination. Embolic materials are carefully introduced into the uterine artery or other feeding arteries until stasis of flow is confirmed angiographically. Ipsilateral internal iliac angiography is repeated to exclude the possibility of additional feeding arteries, which occasionally, become apparent only after the major feeding artery is occluded. 

Then, the contralateral internal iliac artery and uterine artery are examined in the same manner. Embolization of the contralateral uterine artery is performed because of the possibility of cross-filling, followed by contralateral internal iliac angiography. If bleeding does not stop or the vascular abnormality does not disappear, other feeding arteries, such as the ovarian artery, inferior epigastric artery, or middle sacral artery, should be examined. The treatment is usually successful after one or two sessions (20). 

Various embolization materials have been used in these procedures, including gelatin sponge, coils, isobutyl-2-cyanoacrylate, detachable balloons, thrombin, and polyvinyl alcohol, but most iatrogenic uterine vascular abnormalities can be treated safely and effectively by embolization with pledgets of absorbable gelatin sponge (Gelfoam) (21). Absorbable gelatin sponge pledgets are also the material of choice for the embolization of acquired AVMs. 

The advantages of transcatheter arterial embolization include outstanding success rates, low complication rates, avoidance of surgical risks, and preservation of fertility (13). Moreover, successful cases of post-embolization pregnancy have been reported. The side effects of the procedure, such as low-grade temperature, pain, infection, or symptoms, have been documented. Of these, pelvic pain was the main side effect, even requiring opiate and nonsteroidal analgesia. In addition, the procedure has the expected disadvantage of insufficient embolization, demanding a repeat procedure. Lim *et al* reported that one of their patients experienced buttock and lower-limb claudication, which resolved spontaneously and may be a result of extensive and multiple embolizations in the pelvic vessels (22).

Neurologic deficits affecting the lower limb have been reported previously, and seem to be more commonly associated with the use of liquid embolization materials or very small particles (23). Other serious complications, such as perineal skin sloughing, uterovaginal and recto-vesico-vaginal fistulae, and bladder necrosis, have also been reported in series where the internal iliac arteries have been embolized with cryanoacylate as the embolizing agent (23). Nevertheless, the complications of transcatheter arterial embolization are extremely uncommon, and the complication risk is negligible when it is performed by interventional radiology experts. Whether the failure of embolization is a result of the type of embolic material used, expertise of the intervention radiologist, a regrowth of AVMs or the persistence of inherent factors is unclear at present. This issue may be resolved after more of these cases are reported and analyzed.

Other surgical managements reported less frequently include the coagulation of AVM under hysteroscopic guidance, surgical removal of AVM, laparoscopic bipolar coagulation of uterine vessels, and ligation of the uterine artery (4, 24, 25). Currently, hysterectomy is indicated only for women who do not need fertility preservation, have limited access to medical facilities (as may be the case in some areas in resource-poor countries), or in whom embolization therapy fails. 

Embolization failure has been managed successfully with unilateral uterine artery and ovarian ligament ligation in a 32-year-old woman with postmolar uterine AVM after two failed embolization attempts (25). Such options are useful when uterine preservation is desired. Long-term follow-up after an apparently successful embolization may reveal more failures. Successful results have been reported after a shorter period of follow-up (6 weeks), but only a long-term follow-up will determine the true success rate of these procedures (26, 27). 

In the current case report, the diagnosis of AVM by color Doppler and arteriogram determined the correct approach to management, which was uterine artery embolization. The bilateral internal iliac arteries and uterine arteries angiography were operated, followed by the embolization of both uterine arteries. Meanwhile, differential diagnosis was considered for obviating massive uterine hemorrhage. After the procedure, the patient experienced a mild lower abdominal discomfort that was tolerated without the use of any drugs. A patient follow-up by color Doppler imaging 2 weeks after embolization demonstrated a markedly reduced blood flow. The menstrual cycle of the patient returned to normal one month after the follow-up. These results confirmed that the uterine artery embolization was efficient.

Therefore, uterine AVMs should be suspected in women who present with abnormal uterine bleeding and a medical history of Caesarean section, induced abortion, or D&C and so on. The current case report supports the current literature, which suggests that the transcatheter uterine artery embolization of uterine AVMs is a safe and valid alternative to surgical therapy.
